# Positive Effects of Videogame Use on Visuospatial Competencies: The Impact of Visualization Style in Preadolescents and Adolescents

**DOI:** 10.3389/fpsyg.2019.01226

**Published:** 2019-05-31

**Authors:** Luca Milani, Serena Grumi, Paola Di Blasio

**Affiliations:** Department of Psychology, Catholic University of the Sacred Heart, Milan, Italy

**Keywords:** videogames, spatial cognition, preadolescence, adolescence, visuospatial abilities

## Abstract

Use of videogames (VGs) is almost ubiquitous in preadolescents’ and adolescents’ everyday life. One of the most intriguing research topics about positive effects of VG use is about the domain of visuospatial competencies. Previous research show that training with videogames enables children and adolescents to improve their scores in visuospatial tests (such as mental rotation of shapes and cubes), and that such training could overcome gender differences in these domains. Our study aimed at (1) verifying the positive effects of videogame use in the visuospatial domain both for male and female adolescents and preadolescents and (2) verifying whether the visualization style (2D or isometric 3D) of the VG has an influence about the positive effects of gaming. Six measures of visuospatial competency were administered to 318 preadolescents (mean of age = 13.94 years, range 10–18) prior and after a 3-day training with 2D and 3D Tetris. Results indicate that (1) gaming on the whole has slight positive effects both for males and females in enhancing visuospatial competencies, at least in the short term, and (2) it seems that participants who used the videogame with 2D graphics obtained greater improvements in the mental rotation domain while the participants who used the videogame with 3D graphics obtained greater improvements in the spatial visualization domain. However, a general learning effect between T1 and T2 was measured, which was found regardless of Experimental condition, indicating that the effect of training with videogames can be less relevant than expected.

## Introduction

Visuospatial competencies are that realm of cognitive ability definable as: “[…] skills in representing, transforming, generating, and recalling symbolic, non-linguistic information” (Linn and Petersen, 1985, p. 1482). In general terms, visuospatial competencies can be described as the ability to imagine the appearance of a figure (regular or irregular) when it is rotated in space, and to perceive the spatial relations between objects. Halpern (2000) distinguishes several subdomains in visuospatial competencies: spatial perception, mental rotation, spatial visualization, and spatio-temporal ability. They can be defined as follows:

- Spatial perception – the ability to correctly assess and perceive spatial relationships;
- Mental rotation – the ability to mentally rotate (either 2D or 3D) spatial objects to see how they would look from a different angle or perspective;
- Spatial visualization – the ability to perform multiple mental manipulations of spatial information in order to reach a different configuration of the visual stimulus;
- Spatio-temporal ability –the ability to make accurate judgments regarding the timing and the movement of objects through space.

Visuospatial competencies are a domain that is susceptible to improvement via formal (e.g., school, specific exercise) and informal training (e.g., performing day-by-day activities like driving). As children progress in their development, these skills are very important for understanding and mastering material in the physical sciences, engineering, architecture, medicine, etc., In fact, visuo-spatial skills are central in many everyday tasks (e.g., navigating through 2D and 3D maps, using tools, operating machineries, and driving cars, etc.) and are closely related to academic achievement in STEM (science, technology, engineering, and mathematics) disciplines (cf. Holmes et al., 2008; Wai et al., 2009).

### Visuospatial Competencies and Videogames

Videogames (VGs) are a very common cultural artifact among children and pre-adolescents: while on average gamers are 35 years old, over 27% of players are under 18 years of age Entertainment Software Association [Esa] (2016). As regards prevalence of video gaming in youth, two thirds of 6–17 year old Italians (AESVI – ISPO, 2010) and of 16–19 year old Europeans regularly play videogames (ISFE, 2010).

Videogames often represent the first introduction to information technologies for the new generations. In the developmental psychology literature, videogames have been linked to potential negative effects such as increased aggression due to violent contents (Milani et al., 2015) and risk of addiction (Petry et al., 2014; Milani et al., 2018).

However, since the Eighties and Nineties many authors have started to study the “bright side” of videogames, highlighting positive effects of their use. In this light, early studies about visuospatial competencies and VG use have shown how training with *ad hoc* videogames leads to an improvement in performance on pencil and paper visuospatial tests (Gagnon, 1985; McClurg and Chaillè, 1987; Okagaki and Frensch, 1994; Subrahmanyam and Greenfield, 1994), and possibly lessens gender differences (Halpern, 2000; Ferguson et al., 2008).

Moving from these preliminary results, researchers tried to maximize the ecological validity of the studies by leaving the lab setting and focusing on the effects of training with VGs in more naturalistic settings, using off-the shelf videogames as a training tool. Terlecki et al. (2008) specifically used the computer game Tetris to train undergraduate students in spatial cognition and found that the improvement lasted for months and generalized to other spatial tasks. They found that that the growth of visuospatial skills during long-term training with Tetris was continuous for a whole semester (albeit the growth curve flattened after 12 weeks of training) and that these improvements remained detectable some months after the cessation of the training. De Lisi and Wolford (2002) found similar results in third-graders, also using Tetris as a training videogame. Feng et al. (2007) showed that a 10 h training with (action and non-action) videogames significantly enhanced field-of-view of participants.

A meta-analysis by Ferguson (2007) highlights how the most significant effect detectable in the continued use of videogames (VGs) is improvement in visuospatial competencies both in males and females. As regards the amount of training needed to obtain any improvement, Cherney (2008) has found that even 4 h in total led to significant results.

Ferguson et al. (2008) verified how also habits in videogame use (and not only specific training) are strongly correlated with better visuospatial competencies. In fact, children who habitually use videogames (especially action ones) show better performance in visual memory tasks, independently of gender.

Oei and Patterson (2013) showed that such positive effects extend to adulthood and a large array of VGs (action games, non-action games, puzzles, and strategic games), on multiple devices (handheld, home-based), and that the transfer of cognitive enhancement is more likely to take place when game activities are closely matched with real-world tasks. Regarding the positive effects of gaming with VGs, Green and Bavelier (2012) argue that playing videogames may not only enhance cognitive and attentive performance, but also – to a greater extent –facilitate the learning of new tasks via flexible interaction with a highly motivating environment (such as the one in action videogames). In particular, effects of action videogames can be found in different domains of visuo-spatial competencies (cf. *ibidem*, pp. R202–R204): selective attention in space (i.e., the ability to focus attention on a target and ignore distracting information); selective attention in time (i.e., the ability to select relevant information over time); selective attention to objects (i.e., the ability to track many independently moving objects); attentional control (i.e., the ability to flexibly allocate attentional resources); and sustained attention and impulsivity (i.e., the ability to maintain attention active and focused attention while refraining from impulsive reactions).

Granic et al. (2014) argue that playing videogames is beneficial in at least four domains, fostering cognitive, motivational, emotional, and social benefits. As regards the cognitive domain, which is of interest for our paper, Granic et al. argue that spatial skills improvements obtained via commercial videogame play could be comparable to the effects of formal education targeted to the same set of skills, as also reported in the meta-analysis by Uttal et al. (2013). However, not all games are equally effective in this domain, as the action/shooter genre seems to convey the most beneficial effects due to their very dense and complex visual representation, while more visually sparse videogames (as adventures) seem to be less effective. The research from Shute et al. (2015) showed that a mixed action/puzzle commercial videogame such as Portal 2 (which provides compelling puzzles to be solved in order to progress in a first-person complex three-dimensional environment) was more effective in improving visuospatial abilities than a commercial cognitive training program (“Lumosity”).

Beneficial cognitive effects of training with VGs are not limited to the “typical” population but are thought to extend to some atypical populations such as children with learning disabilities. In this area, Franceschini et al. (2013) showed that training with action videogames can improve attention skills, which in turn can improve the reading skills of children with dyslexia. They argue that videogames can represent “low-resource-demanding early prevention programs that could drastically reduce the incidence of reading disorders” (*ibid*, p. 465).

Finally, in terms of “real-world” applications of the potential benefits of training with VGs, one of the most intriguing lines of research deals with the effect of gaming expertise and/or training on trainees in surgery. The seminal work from Rosser et al. (2007) showed that surgeon trainees that never played videogames were the least performant in a laparoscopic training course, while surgeons that played 0–3 h a week were more able than the former, while surgeons that played more than 3 h a week were the most proficient with the laparoscope (scoring 42% better than the non-player group). Following up these correlational results with an experimental setting, Adams et al. (2012) found that a training with an action videogame with a traditional console (Xbox) led to a remarkable improvement in laparoscopic abilities of surgeon residents, while the training on the same videogame on a handheld device (Nintendo DS) led to more modest improvements. The residents that used the usual laparoscopic training box showed the least improvement of the three groups. Kennedy et al. (2011), however, showed that the higher proficiency in laparoscopic ability by those surgeon trainees that were gamers, compared to non-gamers trainees, was due more to psychomotor skills than to visuo-spatial competencies. Khatri et al. (2014) also found that previous experience with videogames was not related to higher performance with a VR simulator of orthopedic procedures. Thus, the contribution of expertise and training in videogames to proficiency in medical procedures requiring visuospatial abilities seems to have been established for some specific procedures such as laparoscopy, but not fully supported for other branches of medicine.

To sum up, the literature seem to have established a fairly robust correlation between practice with videogames and enhancement of visuospatial skills. Given the paradigmatic shift seen in the last 20 years in the videogame industry (i.e., the introduction of complex and real-time three-dimensional graphics), assessing the effects of different types of visualization is of some importance. This holds even truer when thinking about the learning potential embedded in videogames: choosing the “right” balance between scenario complexity, ease of use and sense of engagement in the VG could be very important in order to design videogames for learning purposes. At the moment, to our knowledge, no research has contrasted the potential positive effect on visuospatial competencies of 2D vs. 3D graphics, using the same videogame as a training. Moving from these assumptions, our exploratory research has specific one hypothesis (H1) and a general research question (RQ1):

(H1) Given the positive effects of videogame use in the increase in visuospatial competencies discussed in previous research, we hypothesize that participants in the Experimental groups (2D and 3D Tetris) will show a significant increase in visuospatial scores compared to participants in the control group.
(RQ1) We are interested in assessing whether the graphics of the videogame used (2D or 3D) tap different sub-components of visuospatial competencies and thus affects visuospatial competencies in a different manner. The following RQ is thus posited: Do the graphics (2D vs. 3D) affect visuospatial competencies differently?

## Materials and Methods

### Participants

A total of 318 pre-adolescents and adolescents aged 10–18 years old (mean = 13.94 years old, *SD* = 2.21) were recruited from secondary schools in the Province of Milan. A hundred and sixty participants were male (mean age = 14.09 years, *SD* = 2.29) and 139 were female (mean age = 13.76 years, *SD* = 2.11). In terms of socio-economic status (SES), all participants were middle class. Mean age was not different across gender (*t* = 1.32; n.s.).

Participants were randomly assigned to three experimental conditions: training with a 2D videogame (*N* = 116), training with a 3D videogame (*N* = 119) and control (*N* = 83). The experimental conditions were balanced for gender (χ^2^ = 0.04; *df* = 2; n.s.) and for mean age (*F* = 2.91; n.s.).

### Procedure

Principals of the schools approved the schools’ participation in the research project, agreed to the collection of data and informed the parents about the research. The day before the data were collected, the researchers explained the research to the students in the classroom and gave them an envelope to give to their parents. The envelope included a description of the research and its aims and a consent form to be signed by both parents prior to the administration of the instruments. The following day, the experimenters collected signed informed consent forms and administered the first survey only to students whose parents signed the informed consent. The research was organized into three phases:

T1 – we administered a battery of visuospatial paper tests to the adolescents, together with a specific questionnaire to measure videogame use habits. The questionnaire regarding gaming habits included 20 items with different response formats (9-point Likert scales, open-ended questions, and closed questions). Six subscales of the “Kit of Factor Referenced Cognitive Tests” (Ekstrom et al., 1976) were employed as visuospatial tests. Each of the six subscales consisted of a different visuospatial time-limited task. The time allowed for the completion of the tests ranged from one to 3 min according to the instructions provided in the Manual of the test. The administration of the instruments took place in the classes.

Training Phase – at the end of the administration of the visuospatial tests, 2D and 3D versions of Tetris were distributed via *Dropbox* access codes. The videogames used were “Tetrix2000” for the 2D version of the game and “3DBlocks” for the three-dimensional version. The games were free-distribution games that could run on almost any PC configuration due to low hardware requirements. Moreover, the blocks were the same shape and color in both 2D and 3D versions. The participants were asked to play the videogame at home for 45 min per day on the three following days. The participants were specifically asked not to use any videogame except the one provided by experimenters. Participants in the control sample were asked not to use any videogame for the 3 days. In order to maximize compliance with the instructions, we asked participants to collect a brief diary of their sessions in terms of points achieved for each game and number of games played per session. We opted for a short distributed training setting rather than a single session of mass training, as it seems to be effective and yield durable results (cf. Eichenbaum et al., 2014).

Tetris was selected for different reasons: first of all, it provides a challenging environment, mixing features from puzzle and action VGs; secondly it does not convey any violence nor aggressive content, and – finally – the Tetris learning curve is easy while maintaining a good challenge due to the constant increase in the speed of falling of the bricks. Spence and Feng (2010) assess “puzzle” videogames as having a “medium” effect on visuospatial competencies (albeit inferior to action videogames).

T2 – at the end of the 3 days, we administered an alternative version of the same visuospatial test battery to all participants, in order to hinder any recognition of the correct answers and partially control for learning effects. At the end of the experiment, all participants were granted free access to both versions of Tetris.

### Instruments

The test used to measure visuospatial competencies was composed of six subscales of the “Kit of Factor Referenced Cognitive Tests” (Ekstrom et al., 1976), which covers some of the domains of visuospatial competence highlighted by Halpern (2000). The subscales’ scores were computed as the total number of correct answers for each task, except for Rotation of figures (S-1) and Rotation of cubes (S-2), where scores were corrected against guessing by subtracting the number of errors from the number of correct answers, following Manual guidelines.

The tests were administered to all participants in the following order:

• Dot matrix (CF-3): the task consists in copying a geometrical configuration using a matrix of dots as reference. It measures the capacity to preserve a visual configuration mentally and thus covers Halpern’s spatial perception. Scores could range from 0 to 32.
• Recognition of identical figures (P-3): the task requires recognition of which of the five symbols presented is identical to the one supplied as a model. It measures speed in perceiving and comparing visual stimuli, thus covering Halpern’s spatial perception. Scores could range from 0 to 24.
• Reconstruction of images (CS-1): the task consists of guessing the identity of an object shown incompletely. It measures the capacity to perceive a gestalt from partial data (this covers Halpern’s spatial visualization). Scores could range from 0 to 10.
• Labyrinth (SS-1): the task requires drawing a trail through a labyrinth with many dead-end paths. It also covers the capacity for spatial perception. Scores could range from 0 to 24.
• Rotation of figures (S-1): the task requires deciding whether the figures presented as stimuli are rotations of the model figure. It measures speed in perceiving bi-dimensional spatial relations, thus covering Halpern’s mental rotation. Scores could range from −80 to 80.
• Rotation of cubes (S-2): the task consists of deciding whether the pairs of cubes presented as stimuli are the same cube rotated or different cubes. It measures speed in perceiving tri-dimensional spatial relations, also covering Halpern’s mental rotation. Scores could range from −21 to 21.

Cronbach’s Alpha for the tests (each was considered as an item) was 0.63.

### Data Analysis Strategy

We performed firstly a set of descriptive analyses. Then multilevel modeling was run by means of Generalized Linear Mixed Models (GLMM) nesting Time measurements (Level 1) within participants (Level 2).

A linear model with identity as a link function was selected. Experimental condition (control, 2D, 3D), Time (Time 1, Time 2), and their interaction were included in the model as fixed factors, while Participant was included as a random effect to control for variance due to differences among participants. Bonferroni adjusted pairwise comparisons were employed to analyze differences among conditions. Gender and age were included as covariates. We performed tests of six models, one for each of the visuospatial tests administered.

## Results

### Descriptive Results

Regarding the data derived from the questionnaire on videogame use habits, 98.1% of the participants declared that they played videogames habitually, and the mean of hours per week of videogame play was 5.88. Regarding videogame play, gender differences emerged: while males on average played videogames for 7.97 h a week, females played videogames for only 3.51 h (*t* = 7.99; *p* < 0.001). Mean scores obtained in the six visuospatial subscales administered were compared on the basis of gender. Contrary to the usual findings in the literature, no gender differences were found except in the labyrinth test (males mean = 11.82, female mean = 10.86; *t* = 2.33; *p* < 0.05). On the basis of previous literature and this result, gender was inserted as a covariate in the subsequent analyses.

Mean scores on the six visuospatial tests are presented in Table 1.

**Table 1:** Means and Standard Deviations of scores in the six scales of visuospatial competence by three experimental conditions.

	Experimental condition					
	2D		3D		Control	
	T1	T2	T1	T2	T1	T2
Dot matrix (CF-3)	15.57 (5.87)	18.97 (6.58)	15.08 (7.30)	19.03 (7.72)	13.20 (6.76)	15.39 (7.17)
Recognition of identical figures (P-3)	35.95 (7.56)	38.13 (7.09)	33.93 (9.00)	38.39 (7.95)	30.83 (10.29)	34.62 (9.43)
Reconstruction of images (CS-1)	6.79 (1.77)	6.57 (2.11)	6.77 (1.72)	6.44 (2.25)	6.65 (1.36)	6.12 (2.04)
Labyrinth (SS-1)	11.93 (3.80)	15.29 (3.97)	11.45 (3.50)	15.86 (4.24)	10.51 (3.69)	14.24 (3.80)
Rotation of figures (S-1)	38.19 (17.93)	44.76 (18.60)	34.24 (21.59)	38.48 (22.67)	30.72 (21.11)	34.01 (22.92)
Rotation of cubes (S-2)	5.46 (3.90)	4.49 (5.40)	4.95 (5.16)	4.67 (5.29)	4.27 (4.87)	3.34 (5.42)

### Effects of the Use of the Videogame on Visuospatial Competencies

Results of the Generalized Linear Mixed Models are presented in Table 2, while Table 3 presents confidence intervals.

**Table 2:** Linear mixed model: Parameter estimates for the six measures of visuospatial competences.

	Dot matrix (CF-3)	Recognition of identical figures (P-3)	Reconstruction of images (CS-1)	Labyrinth (SS-1)	Rotation of figures (S-1)	Rotation of cubes (S-2)
Measure/Parameter	Est (SE)	Est (SE)	Est (SE)	Est (SE)	Est (SE)	Est (SE)
**Fixed effects**						
Intercept	14.33^∗∗∗^ (0.635)	31.98^∗∗∗^ (0.923)	6.93^∗∗∗^ (0.171)	11.50^∗∗∗^ (0.374)	33.61^∗∗∗^ (2.35)	5.33^∗∗∗^ (0.502)
Exp. condition (2D)	0.870 (0.767)	2.62^∗^ (1.12)	−0.114 (0.208)	0.823 (0.450)	5.16 (2.83)	0.440 (0.606)
Exp. condition (3D)	0.469 (0.753)	1.77 (1.10)	−0.167 (0.204)	0.279 (0.441)	1.24 (2.78)	0.048 (0.595)
Time of measurement	2.25^∗∗∗^ (0.479)	3.78^∗∗∗^ (0.677)	−0.597^∗∗^ (0.208)	3.65^∗∗∗^ (0.318)	3.23^∗^ (1.30)	−1.04^∗^ (0.535)
Time × exp. cond. (2D)	1.55^∗^ (0.628)	−0.320 (0.888)	0.386 (0.271)	−0.267 (0.417)	4.40^∗∗^ (1.71)	0.277 (0.691)
Time × exp. cond. (3D)	1.63^∗∗^ (0.620)	0.689 (0.875)	0.262 (0.267)	0.771^∼^ (0.411)	1.97 (1.68)	0.818 (0.680)
Gender	0.491 (0.568)	0.393 (0.786)	−0.073 (0.148)	−0.818^∗^ (0.341)	−2.57 (2.13)	−0.960^∗^ (0.412)
Age	2.03^∗∗∗^ (0.130)	1.88^∗∗∗^ (0.179)	0.452 (0.033)	0.910^∗∗∗^ (0.077)	3.13 (0.487)	1.20^∗∗∗^ (0.093)
**Variance components**						
Residual (T1)	6.03^∗∗∗^ (1.31)	18.65^∗∗∗^ (2.85)	0.898^∗∗∗^ (0.155)	1.53^∗∗^ (0.498)	46.42^∗∗∗^ (12.96)	8.04^∗∗∗^ (1.15)
Residual (T2)	11.44^∗∗^ (1.53)	16.34^∗∗∗^ (2.76)	2.28^∗∗∗^ (0.234)	6.16^∗∗∗^ (0.701)	82.97^∗∗∗^ (14.22)	12.16^∗∗∗^ (1.38)
**Random effect**						
Subject	19.85^∗∗∗^ (2.04)	36.91^∗∗∗^ (3.83)	0.963^∗∗∗^ (0.156)	7.36^∗∗∗^ (0.774)	307.34^∗∗∗^ (28.21)	7.42^∗∗∗^ (1.11)

*Note: ^∗∗∗^p < 0.001; ^∗∗^p < 0.01; ^∗^p < 0.05; ^∼^p < 0.06. The estimates reported in this table were obtained using Control and Time 1 as reference conditions for Experimental Condition and Time of measurement, respectively.*

**Table 3:** Linear mixed model: Confidence intervals (95%).

	Dot matrix (CF-3)	Recognition of identical figures (P-3)	Reconstruction of images (CS-1)	Labyrinth (SS-1)	Rotation of figures (S-1)	Rotation of cubes (S-2)
**Fixed effects**						
Intercept	13.08, 15.58	30.16, 33.79	6.60, 7.27	10.76, 12.23	28.98, 38.24	4.34, 6.32
Exp. condition (2D)	−0.63, 2.37	0.42, 4.83	−0.52, 0.29	−0.06, 1.70	−0.40, 10.77	−0.75, 1.63
Exp. condition (3D)	−1.01, 1.94	−0.38, 3.94	−0.56, 0.23	−0.55, 1.14	−4.23, 6.71	−1.12, 1.21
Time of measurement	1.30, 3.19	2.44, 5.11	−1.00, −0.18	3.03, 4.28	0.67, 5.79	−2.09, 0.00
Time × exp. cond. (2D)	0.31, 2.78	−2.06, 1.42	−0.14, 0.91	−1.08, 0.55	1.04, 7.71	−1.08, 1.63
Time × exp. cond. (3D)	0.41, 2.84	−1.03, 2.40	−0.26, 0.78	−0.03, 1.58	−1.33, 5.28	−0.51, 2.15
Gender	−0.62, 1.60	−1.15, 1.93	−0.36, 0.22	−1.48, −0.14	−6.76, 1.63	−1.77, −0.15
Age	1.77, 2.28	1.53, 2.23	0.38, 0.51	0.75, 1.06	2.17, 4.09	1.01, 1.38
**Variance components**						
Residual (T1)	3.92, 9.24	13.82, 25.17	0.64, 1.26	0.81, 2.89	26.86, 80.25	6.07, 10.66
Residual (T2)	8.80, 14.87	11.73, 22.77	1.87, 2.79	4.93, 7.70	52.29, 116.09	9.73, 15.19
**Random effect**						
Subject	16.22, 24.27	30.11, 45.25	0.70, 1.32	5.99, 9.04	256.74, 367.92	5.52, 9.97

### Dot Matrix (CF-3)

By looking at estimates, it appears that 2D condition ameliorates control group mean score by 1.55 points while 3D condition edges control group scores by 1.63 points. Confidence intervals range from 0.31 to 2.78 and from 0.41 to 2.84, respectively. Thus, it appears that the training obtained a moderate effect if compared to control group trend from T1 to T2.

### Recognition of Identical Figures (P-3)

Participants in the 2D condition showed a worse performance than participants in the control group by 0.32 points, while 3D condition means are higher than those of control group by 0.68 points. Confidence intervals range from −2.06 to 1.42 and from −1.03 to 2.40, respectively. As regards this specific subscale, it appears that the training with the videogame was not effective from T1 to T2. Results also show high dispersion within experimental conditions, showing that any difference is likely to be related to learning effects from T1 to T2.

### Reconstruction of Images (CS-1)

Participants’ scores in 2D condition were 0.38 points higher than scores of participants in control condition. Scores in 3D condition were 0.26 points higher than controls. Confidence intervals range from −0.14 to 0.91 and from −0.26 to 0.78, respectively. As for P-3, it appears that the training with the videogame was not effective in improving visuo-spatial abilities in this specific subscale.

### Labyrinth (SS-1)

By looking at estimates, it appears that 2D condition scores are lower than scores of control group by 0.27 points, while 3D condition edges control group scores by 0.77 points. Confidence intervals range from −1.08 to 0.55 and from −0.03 to 1.58, respectively. These lead to ascribe a negligible effect of the 3D training and to categorize 2D training as ineffective.

### Rotation of Figures (S-1)

Participants’ scores in 2D condition were 4.40 points higher than scores of participants in control condition. Scores in 3D condition were 1.97 points higher than controls. These parameters lead us to categorize the training with the videogame as effective, especially in the 2D condition. However, confidence intervals range from 1.04 to 7.71 and from −1.33 to 5.28, respectively. This cautions against ascribing the improvement from T1 to T2 to training effects alone, as some learning may be happened between measurements.

### Rotation of Cubes (S-2)

Estimates show that 2D condition scores are higher than scores of control group by 0.27 points, while 3D condition edges control group scores by 0.81 points. Confidence intervals range from −1.08 to 1.63 and from −0.51 to 2.15, respectively. These lead to ascribe a negligible effect of the 3D training and to categorize 2D training as ineffective.

## Discussion

Results of this exploratory research indicate, first of all, that participants performances increased between T1 and T2 in many of measures administered, regardless of Experimental condition. In particular, participants performances were better at T2 in the following subscales: Dot Matrix (CF-3), Recognition of identical figures (P-3), Labyrinth (SS-1) and Rotation of figures (S-1). This indicates a very likely effect of learning occurring between T1 and T2, implying the need for a prudent approach about the effects of training with videogames on visuospatial competencies. Literature shows that learning effect is a common artifact in cognitive testing (cf. Bartels et al., 2010).

For two of the tests (Reconstruction of images [CS-1] and Rotation of cubes [S-2]), participants showed worse performance at T2 than at T1. In our opinion, this could be due to a) the nature of the task of reconstructing images, which is not likely to be affected by learning since the figures in the T2 task were different than those in T1, and b) the intrinsic difficulty of the task relating to rotation of cubes. As regards the latter, in particular, the task is more suitable for older children and young adults than younger children, since it requires deciding whether the rotated test cube matches the model cube by evaluating whether the symbols on the faces of the cube can be compatible given the rotation.

As regards the main RQs of our study, we can conclude that for some of the subscales administered, the use of the videogame was seemingly effective for enhancing visuospatial competencies, at least in the short term. The participants that played Tetris slightly improved their performance compared to those in the control condition. Training with the videogame, according to our data, seems to be marginally effective in enhancing visuospatial abilities in some of the tests administered (H1). In particular, participants in the 2D Experimental condition obtained better scores than participants in the control condition in the Dot matrix (CF-3) and Rotation of figures (S-1) tests, while participants in the 3D Experimental condition showed higher scores in the Dot matrix (CF-3), Labyrinth (SS-1) and Rotation of Cubes (S-2) tests than participants in the control condition. On the whole, these results are likely to indicate that even a brief training on VGs can likely tap into Halpern’s spatial perception and mental rotation visuospatial subskills. However, spatial visualization was not affected by the training.

We attribute these results to the fact that Tetris seem to match specifically both spatial perception – in terms of perceiving and assessing spatial relations between the shapes and their relative position according to the boundaries of the “well” – and mental rotation – in terms of quickly assessing the (either 2D or 3D) rotation needed to fit the pieces together. These results mirror previous research that found similar differential effects (Terlecki and Newcombe, 2005; Terlecki et al., 2008). On the contrary, spatial visualization seemed to be relatively unaffected by the training. We attribute this result to the mismatch between visuospatial operations performed in Tetris and the specific visuospatial task (CS-1), which required guessing figures of well-known objects (e.g., a ship) from an incomplete model figure.

As regards age, the results seem to indicate that in four of the tests older participants outperformed younger participants; in particular: Dot matrix (CF-3), Reconstruction of images (CS-1), Labyrinth (SS-1) and Rotation of cubes (S-2). Previous research reports that age is related to better visuospatial skills (Del Giudice et al., 2000; Rosselli and Ardila, 2003), so this is a non-surprising result. Moreover, since age was inserted as a covariate in all the models tested, we can be confident that any effect of VG practice detected in our study can be thought of as occurring independently of age. Also Gender was found to potentially influence some of the tests: Labyrinth (SS-1) and Rotation of cubes (S-2). As in the case of age, some research highlighted gender differences in visuospatial skills (cf. Halpern, 2000, for a review), so we believe this result is quite expected. Having inserted Gender as a potential covariate, we believe that any effect of VG practice on visuospatial competencies can be considered to be independent of gender.

In terms of visualization differences (cf. RQ1), it seems that participants who used the videogame with 2D graphics obtained greater improvements than those who used the videogame with 3D graphics in the domain of mental rotation of figures (S-1). On the contrary, participants who used the videogame with 3D graphics did not show higher scores in spatial visualization (S-2). The experimental conditions seemed to be equally effective in promoting some slight improvement in spatial perception (CF-3 test). It seems that the somewhat limited training time with Tetris conferred some positive effects for some visuospatial abilities, but not all of them. In particular, it seems that spatial perception and mental rotation are more sensitive to this type of training than spatial visualization. We attribute this result mainly to the intrinsically higher complexity of the spatial visualization subskill (it requires multiple mental manipulations of spatial information), which probably means that a longer training time would be needed to detect any improvement. We also attribute these results to the fact that the tasks that the scales we found to be slightly improved by training with the videogame are probably the most similar to the game play of Tetris: rapid scanning of the visual space (CF-3, Dot matrix), movement in the X- and Y- axes (SS-1 Labyrinth) and rotation of bricks (S-1, Rotation of figures). Generalizing these results, it seems that – given the limited time spent in training with the videogames we provided – the training on the videogames could have had the role of a “trigger” that probably allowed the participant to recover previous abilities probably related to the habits of videogame use (all participants indicated that they played VGs). The literature in fact shows that more than intensive training, it is the frequent VGs that has a substantial effect in improving cognitive competencies linked to the spatial domain (Green and Bavelier, 2007; Cherney, 2008; Ferguson et al., 2008). We believe these effects should be taken cautiously, however, since the performances in the visuospatial tests were also characterized by a large amount of variability among participants.

These results as a whole could have some implications for educational software development. If we think about the growing educational potential of videogames in formal and informal education, results of our study could be of some use to aiding in the design of computerized training programs for disadvantaged children (cf. Demily et al., 2016), in particular in identifying the most appropriate graphic visualization style for addressing specific visuospatial abilities.

### Limitations of the Research and Future Directions

The main limitation of this research, which is exploratory in its nature, is the limited control over the training sessions due to the home setting. This allowed the research to be carried out in a more ecologically valid setting and led to interesting results; however, we could not control compliance with the instructions, nor the equipment used to play the games. Unfortunately, we had to conform to setting constraints (i.e., some schools did not have enough computers for each participant and not enough time frames for the training during school hours), so we opted for a home setting to overcome these restrictions.

A second limitation of the research lies in its design. A mixed cross-sectional and longitudinal methodology would have allowed us to test not only the improvement effect of the training with the videogame on visuospatial competencies, but also the persistence of this effect.

A further limitation regards the pen-and-paper nature of the visuospatial tests used. As research by Okagaki and Frensch (1994) shows, the accuracy of the measurement in the visuospatial domain seems to be higher when the tests administered are formally consistent with the nature of the training.

A fourth limitation could regard the device where the VGs were played: PCs are just one of the options available and future research should differentiate the devices to assess any differences related to interaction (i.e., touch vs. keyboard) and screen dimension (i.e., PC monitor vs. tablet vs. smartphone). Future lines of research will need to generalize results of this PC-based research on different devices and settings.

Finally, a fifth limitation lies in the training time we were allowed to involve participants in, as it was several hours below the usual training time usually found in the literature. We believe that the small effect of the training with videogames we found on visuospatial competences can be mainly ascribed to this limitation. Nonetheless, the fact that, in some of the visuospatial tasks, the training was apparently effective in promoting a slight improvement can be read also as a testimony of the potentials of using videogames as a training tool. Overall, this exploratory research has shown a slight effectiveness of the training with videogames on some visuospatial competencies, at least in the short term, together with some differential effects of 2D vs. 3G graphics on subcomponents of these competencies. However, these preliminary results need to be replicated in more controlled experimental environments and with bigger samples, and to generalized to more ample age groups before they can be established as robust.

## Ethics Statement

An ethics approval was not required at the time the study was conducted as per the Università Cattolica del Sacro Cuore’s guidelines and national regulations. Written informed consent was obtained from the parents of all participants, and our study is compliant with Helsinki Declaration principles as regards research with humans, even in the absence of a formal Ethic Committee evaluation.

## Author Contributions

LM conceptualized the study, collected the data, performed statistical analyses, and wrote the first draft. SG contributed in performing statistical analyses and edited the manuscript. PDB conceptualized the study and edited the manuscript.

## Conflict of Interest Statement

The authors declare that the research was conducted in the absence of any commercial or financial relationships that could be construed as a potential conflict of interest.
